# A Manual of the Physical Diagnosis of Thoracic Diseases

**Published:** 1888-04

**Authors:** 


A MANUAL OF THE PHYSICAL DIAGNOSIS OF THORACIC DISEASES. By E. Darwin Hudson, Jr., A. M., M. D., late Professor of General Medicine in the New York Polyclinic. New York: William Wood & Company.

The death of Dr. Hudson last year cut short a remarkably successful career. Both as a teacher and as a practitioner he had attained considerable eminence, although still a young man. Fortunately for the profession, the present work was completely finished ere death overtook him. It is the last and best contribution of his pen, and will undoubtedly take rank with the best works on physical diagnosis. A feature of the work which will especially commend it to students is the inclusion of tables of synopses of thoracic diseases, thereby enabling the reader to refresh his memory on the subjects of symptoms, pathology, treatment, etc., while learning the physical signs. The book is profusely illustrated with very good cuts.

